# An unusual presentation of acute myocardial infarction in physiotherapy direct access: findings from a case report

**DOI:** 10.1186/s40945-021-00099-x

**Published:** 2021-02-15

**Authors:** Lorenzo Storari, Valerio Barbari, Fabrizio Brindisino, Marco Testa, Maselli Filippo

**Affiliations:** 1 Department of Neurosciences, Rehabilitation, Ophthalmology, Genetic and Maternal Infantile Sciences (DINOGMI), University of Genova - Campus of Savona, Savona, Italy; 2 Department of Medicine and Health Science “Vincenzo Tiberio”, University of Molise c/o Cardarelli Hospital, C/da Tappino, Campobasso, Italy

**Keywords:** Anterior wall myocardial infarction, Differential diagnosis, Referral and consultation, Shoulder pain, Physiotherapy

## Abstract

**Background:**

Shoulder pain (SP) may originate from both musculoskeletal and visceral conditions. Physiotherapists (PT) may encounter patients with life-threatening pathologies that mimic musculoskeletal pain such as Acute Myocardial Infarction (AMI). A trained PT should be able to distinguish between signs and symptoms of musculoskeletal or visceral origin aimed at performing proper medical referral.

**Case presentation:**

A 46-y-old male with acute SP lasting from a week was diagnosed with right painful musculoskeletal shoulder syndrome, in two successive examinations by the emergency department physicians. However, after having experienced a shift of the pain on the left side, the patient presented to a PT. The PT recognized the signs and symptoms of visceral pain and referred him to the general practitioner, which identified a cardiac disease. The final diagnosis was acute myocardial infarction.

**Conclusion:**

This case report highlights the importance of a thorough patient screening examination, especially for patients treated in an outpatient setting, which allow distinguishing between signs and symptoms of musculoskeletal from visceral diseases.

## Background

Shoulder pain (SP) is one of the most common musculoskeletal (MSK) disorders in the general population with a prevalence ranging from 7 to 27% among adults younger than 70 years-old [1]. SP is the third most common MSK disease after lower back and neck pain, furthermore, it is also one of the most prevalent complaints in outpatient clinic [1, 2], and in emergency department (ED) [3, 4]. Most of the patients consulting healthcare professionals for SP (in primary and secondary care) have been diagnosed with subacromial impingement syndrome, rotator cuff tendinopathy and adhesive capsulitis [1, 5, 6]. However, in addition to the musculoskeletal or mechanic origin of pain, healthcare professionals must be alert that visceral [7], or serious potential life-threatening illnesses may refer pain to the shoulder, such as cardiovascular disorders [7–9]. In fact, patients with cardiovascular pathologies may present crushing, substernal chest pain, abdominal pain, jaw pain, neck pain, diaphoresis, dyspnea, fatigue and arm or shoulder referred pain as main signs and symptoms [8, 9], with some differences given by sex [8].

Among cardiovascular pathologies, high rates of both incidence and prevalence are represented by acute myocardial infarction (AMI )[10, 11, 11], which most common sites of pain are chest, upper limb and abdomen [12, 13]. Unfortunately, these possible referred pain locations could lead to misleading diagnosis even in ED context [14]. Therefore, healthcare professionals, including physiotherapist (PT), should evaluate each possible underlying cause during the evaluation of patients referring SP [9, 15, 16]. Currently, there is sparse literature regarding cases of differential diagnosis made by PT in patients with musculoskeletal diseases [17–19], especially in case of cardiogenic referred pain [20]. Moreover, it is not available at present any reports related to an acute life-threatening condition such as AMI. We report the case of a patient presented to an outpatient physiotherapy clinic with SP that quickly shifted from the right to the left shoulder, which has been properly recognized as visceral related pain by the PT, and finally referred to an appropriate medical management.

## Case presentation

**Fig. 1:**
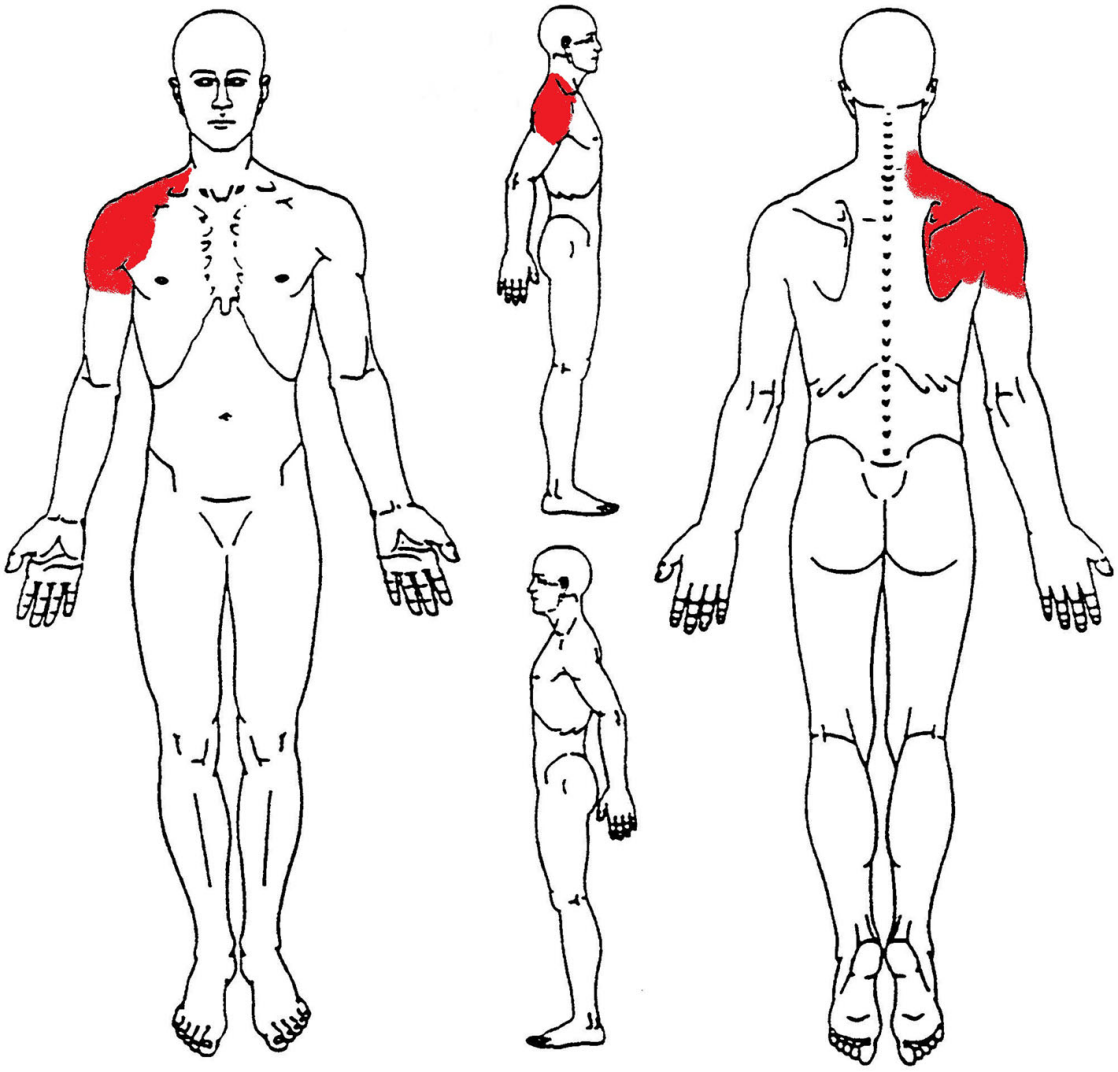
Body chart. Symptoms at 1st ED visit. In red NPRS 8/10 at rest

A 46-y-old tiler male presented to the ED complaining of right SP during the previous week due to increased workload (13–14 h per day laying bulky tiles, instead of his usual 8 h of daily work). His vitals parameters were pulse oxygen saturation level of 99%, blood pressure (BP) of 130/90 mmHg, respiratory rate (RR) of 16 rpm, and heart rate (HR) of 72 bpm. Cardiopulmonary and neurological examinations were unremarkable. The patient denied family history for cardiovascular disease, any medication intake in the previous 48 h, and declared being smoker since 20 years. Physical examination documented worsening of pain on palpation of the acromial and scapular region stated with numeric pain rating (NPRS) scale at 8/10, and there were no other abnormalities in other physical examinations (Fig. 1). The pain was described as dull and intense, lasting from a week, with a subtle onset. He was diagnosed by the ED physician with right painful musculoskeletal (MSK) shoulder syndrome and treated with an intramuscular injection of diclofenac 75 mg/mL and ketorolac 30 mg/mL, with no relief. Accordingly, to reduce the pain and calm down the patient, the physician prescribed an infusion of saline solution (100 mL and 250 mL) and ranitidine hydrochloride 50 mg/5 mL and a diazepam dose 10 mg/ 2 mL, which led to a sensible improvement of the pain.

Upon discharge, the patient was advised to take eperisone hydrochloride 100 mg twice and ibuprofen 600 mg twice a day for 5 days, rest from work for 1 week and a prescription of antalgic electrotherapies.

The patient decided to seek his PT to have another evaluation. On examination, he arrived at the clinic holding his upper limb in an antalgic posture, with his arm adducted and internally rotated using a foulard knotted behind the neck. His past medical history included frequent recurrent right shoulder dislocations in the childhood, which had been resolved with muscle strengthening, and chronic gastritis (treated with antacid drugs when needed). The patient denied any other past or current traumas or diseases. He also denied general constitutional symptoms, any unexplained recent weight loss or gain, or bowel or bladder symptoms. He reported smoking 30 cigarettes per day, a body mass index of 27,6 kg/m^2^ and denied any congenital or family history of heart diseases. Disability scored 56.57 on the QuickDASH scale [21]. The physical assessment of the shoulder documented pain on the palpations of the upper trapezius, supraspinatus, infraspinatus, anterior and posterior deltoid muscles with reproduction of symptoms, NPRS =8/10. Hence, vital parameters were not evaluated [13, 15, 22, 23].

Passive range of motion (ROM) of the shoulder was evaluated. Elevation and abduction were both limited to 70 degrees with an empty end-feel [24]. Internal rotation had a painful capsular end-feel and full ROM, whereas external rotation and extension had full ROM and pain-free capsular end-feel. Due to the functional limitation, the abduction and adduction movements on the horizontal plane were performed at 70 degrees of elevation. The abduction had a capsular end-feel while adduction had a tissue approximation end-feel, both pain-free. Active ROM movements of the right shoulder were requested: elevation and abduction were both limited to 70 degrees due to sharp pain in the whole deltoid region, while extension was normal. Due to the pain, external rotation had been performed between 0° and 70° degrees of elevation and were normal, as well as internal rotation, abduction and adduction on the horizontal plane. Manual isometric muscle tests were accomplished for the rotator cuff muscles, deltoid, trapezius, biceps brachialis, triceps and pectoralis major and minor, in accordance with muscle testing usual practice [25], reporting strength comparable to that of the left shoulder (4/5 level on the medical research council scale [MRC] )[26]. To complete the physical examination, active and passive movements of the cervical spine were evaluated [25, 27–29] and were within normal ranges, also upper limb neurodynamic tests were found normal [30, 31]. Due to high irritability, absence of trauma or relevant risk factors for specific shoulder orthopedic pathologies (e.g. superior labral tear from anterior to posterior (SLAP) lesions or full thickness rotator cuff tears), the PT decided not performing passive or orthopedic special tests [32, 33]. For these reasons, according to the diagnostic triage proposed by Ristori et al .[34], diagnosis was non-specific SP on supposed peripheral nociceptive pain mechanism. At the end of the physiotherapy session, the patient was instructed to perform self-mobilization of the shoulder as home exercise twice a day for 3 days until the next physiotherapy treatment [35, 36].

After the physiotherapy session, the patient experienced relief of the symptoms until the same evening, NPRS = 5/1 0[37]. Then, the pain has reoccurred and increased, till forcing him to come back to the ED. The ED physician carried out another medical examination. Vital parameters at the time of admission were BP of 110/90 mmHg, RR of 16 rpm, HR of 98 bpm and pulse oximetry saturation level of 100%. Any other type of clinical assessment was unremarkable. The conclusive diagnosis was right MSK shoulder syndrome, and the ED physician performed an intramuscular injection of thiocolchicoside 4 mg/ 2 ml and diclofenac 75 mg/ 3 ml for pain-relieving. Upon discharge, the patient was instructed performing a daily intramuscular injection with the same dosages and drugs.

## Differential diagnosis

**Fig. 2:**
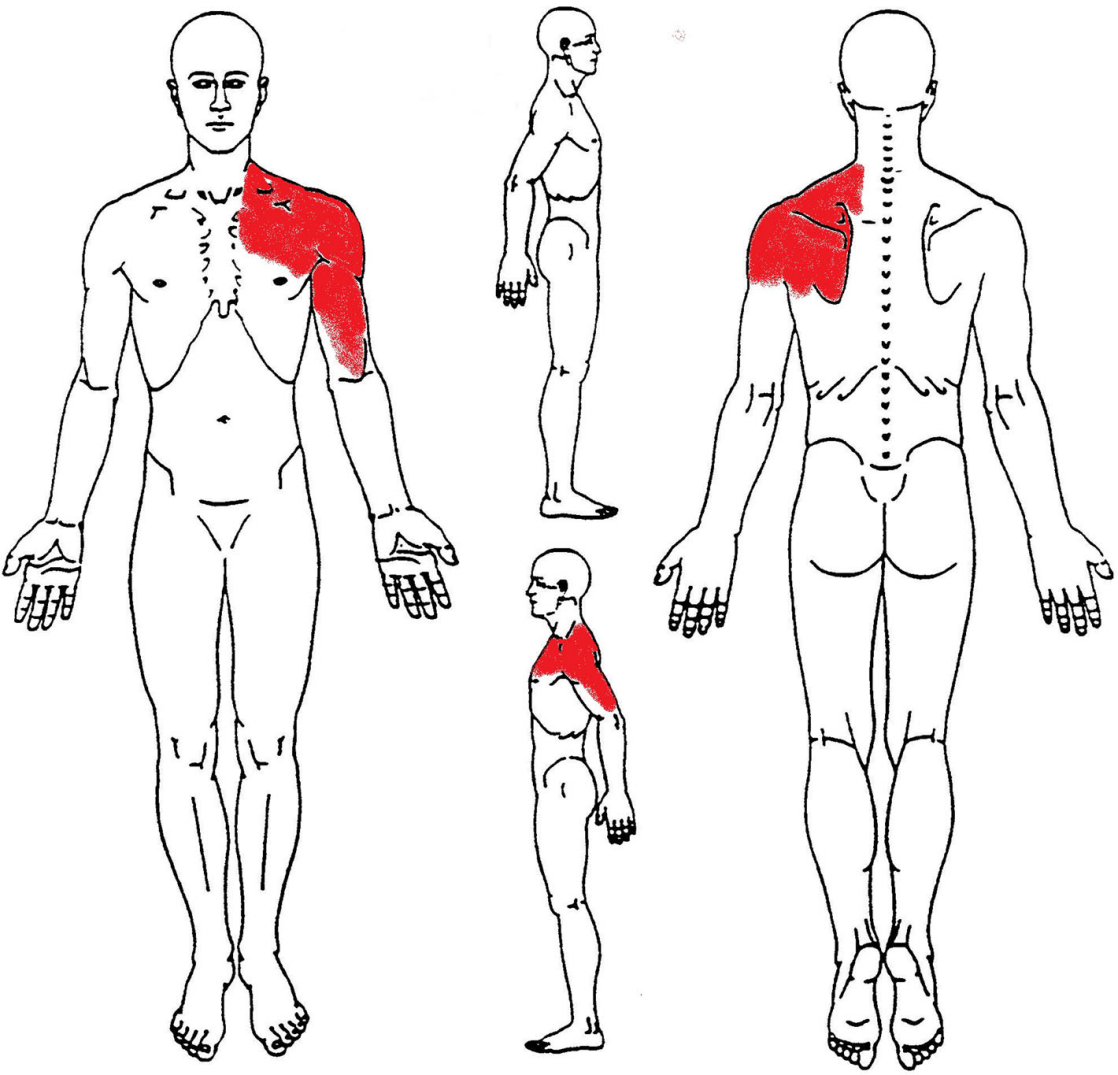
Body chart. Symptoms at 2nd PT visit. In red NPRS 9/10 at rest

The morning of the day after, the patient returned to the outpatient physiotherapy’s clinic arguing that pain had shifted from the right shoulder to the left (Fig. 2). New symptom was sharp pain on the left shoulder, described as constant and oppressive (NPRS = 9/10), radiating down the upper limb and the pectoral region, while the right SP was currently improved at NPRS = 4/10. The patient also reported systemic symptoms as dyspnea, fatigue, shortness of breath and diaphoresis, while palpation of the painful regions did not reproduce his own symptoms [15, 22, 38]. Examinations of cervical and thoracic spine, as well as costochondral joints were found normal [39]. Therefore, the PT correlated the current new symptoms to an underlying cardiovascular referred pain [40, 41]. In order to properly screen possible red flags, the OSPRO-ROS 23-item review-of-systems screening tool [42–45] was administered with positive response, proving the need of a more focused review-of-systems. Vitals were evaluated using automatic monitoring devices as blood pressure machine, SFIGMO DIGITALE AUTOMATICO-SMART (mod.32921, GIMA S.p.A, 20060, Gessate (Milan),Italy), and index finger oximeter, OXY-3 (mod.35090, GIMA S.p.A, GIMA S.p.A, 20060, Gessate (Milan),Italy), reporting BP of 135/90 mmHg, HR of 95 bpm, pulse oxygen saturation level of 97%, and RR of 25 rpm. Furthermore, the PT decided to refer him to his general practitioner, which after having performed a detailed review-of-systems and cardiopulmonary auscultation sent the patient to the ED with a suspected AMI.

On admission at the ED, the patient underwent a third medical assessment. His vitals were BP of 130/90 mmHg, HR of 98 bpm, RR of 25 rpm, pulse oximetry saturation level of 97%. Neurologic and pulmonary systems were normal and abdominal palpation revealed minimal tenderness in the epigastric region.

**Fig. 3:**
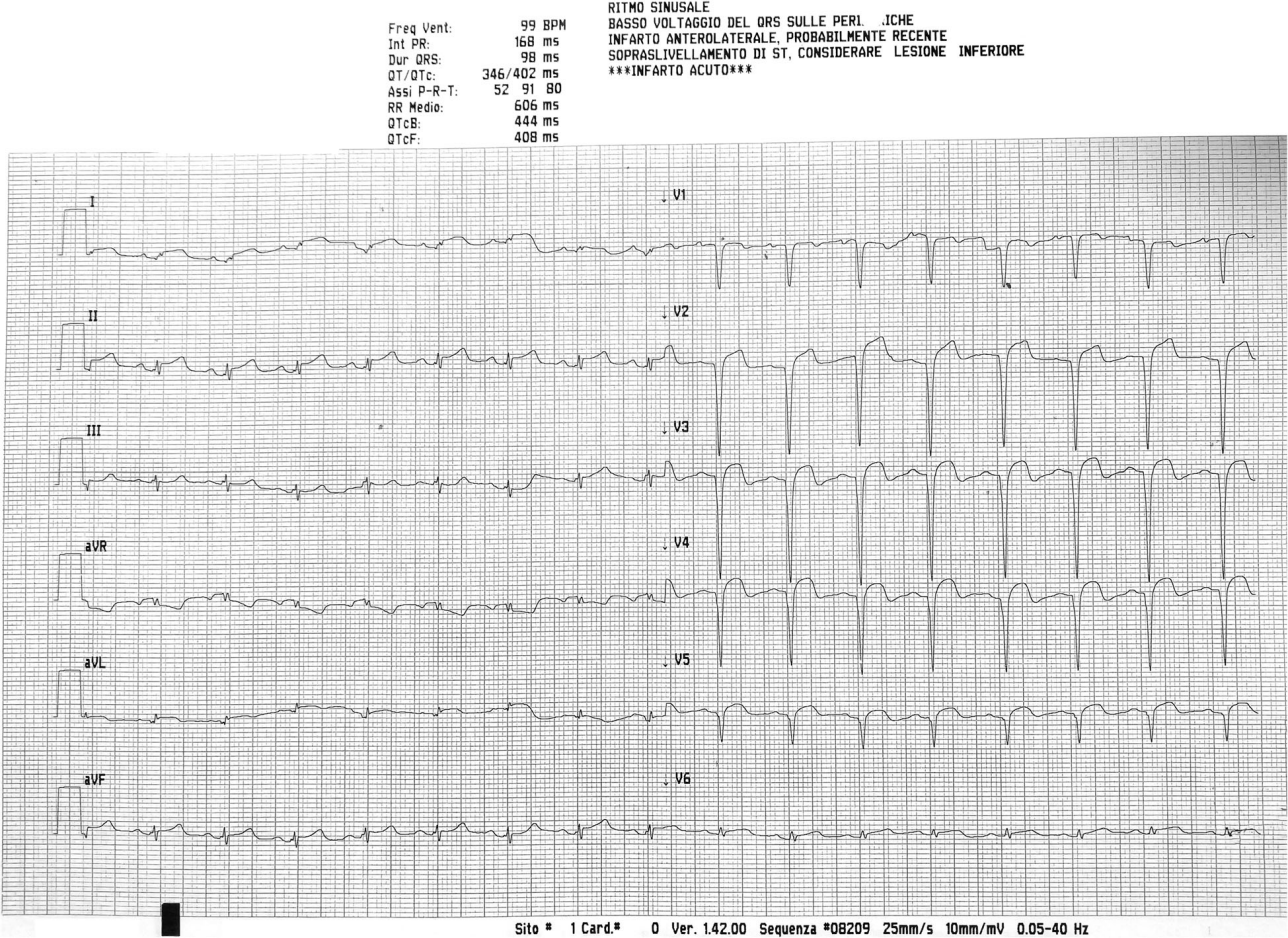
ECG: Sinus Rhythm, 85 bpm, pathological-Q waves in leads I, aVL and the precordial lead V4 with ST-segment elevation in the corresponding leads, ST-segment depression in DIII (anterolateral myocardial infarction)

During the cardiologic visit 12-lead electrocardiography (ECG) and routine blood test were performed. The ECG showed elevation of the ST-segment and low voltage of the QRS complex (Fig. 3).

**Fig. 4:**
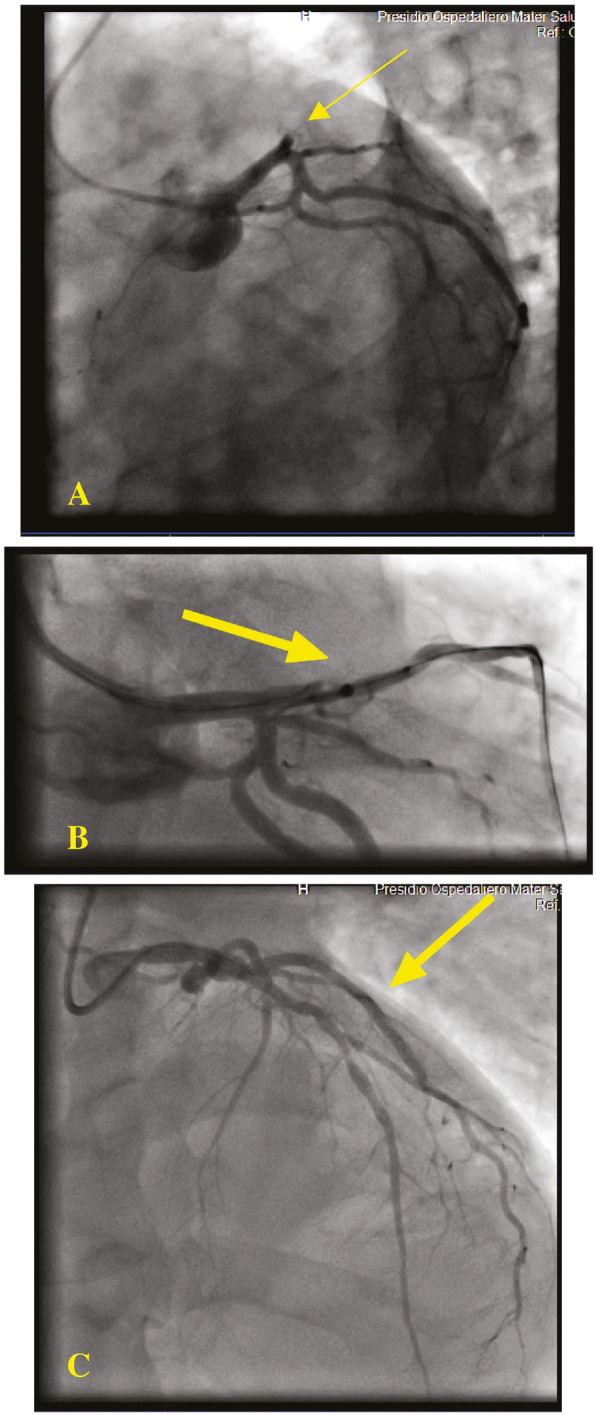
LAD flow showed by coronary angiography with contrast dye. **a** Thick yellow arrow indicates percutaneous transluminal coronary angioplasty, with catheter system that it is introduced through a systemic artery under local anesthesia a stenotic LAD coronary artery. **b** Thick yellow arrow indicates dilate the stenotic artery by controlled inflation of a distensible balloon. **c** Thick yellow arrow indicates the disappearance of stenosis and artery flow restoration

High level of cardiac troponin 4.9 mg/dL was detected. Afterwards bedside echocardiogram and color Doppler showed anterolateral septal-apical necrosis, severe left ventricular disfunction and light pulmonary arterial hypertension. Lastly, coronary angiography demonstrated a complete occlusion of proximal portion of left anterior descending artery (LAD) (Fig. 4). The patient was admitted to the intensive cardiologic care unit with a diagnosis of acute ST-elevation myocardial infarction due to occlusive coronary artery disease. Percutaneous transluminal coronary angioplasty (PTCA) was performed and a drug-eluting stent (DES) on LAD was planted in the cath lab immediately after.

## Outcomes

**Fig. 5:**
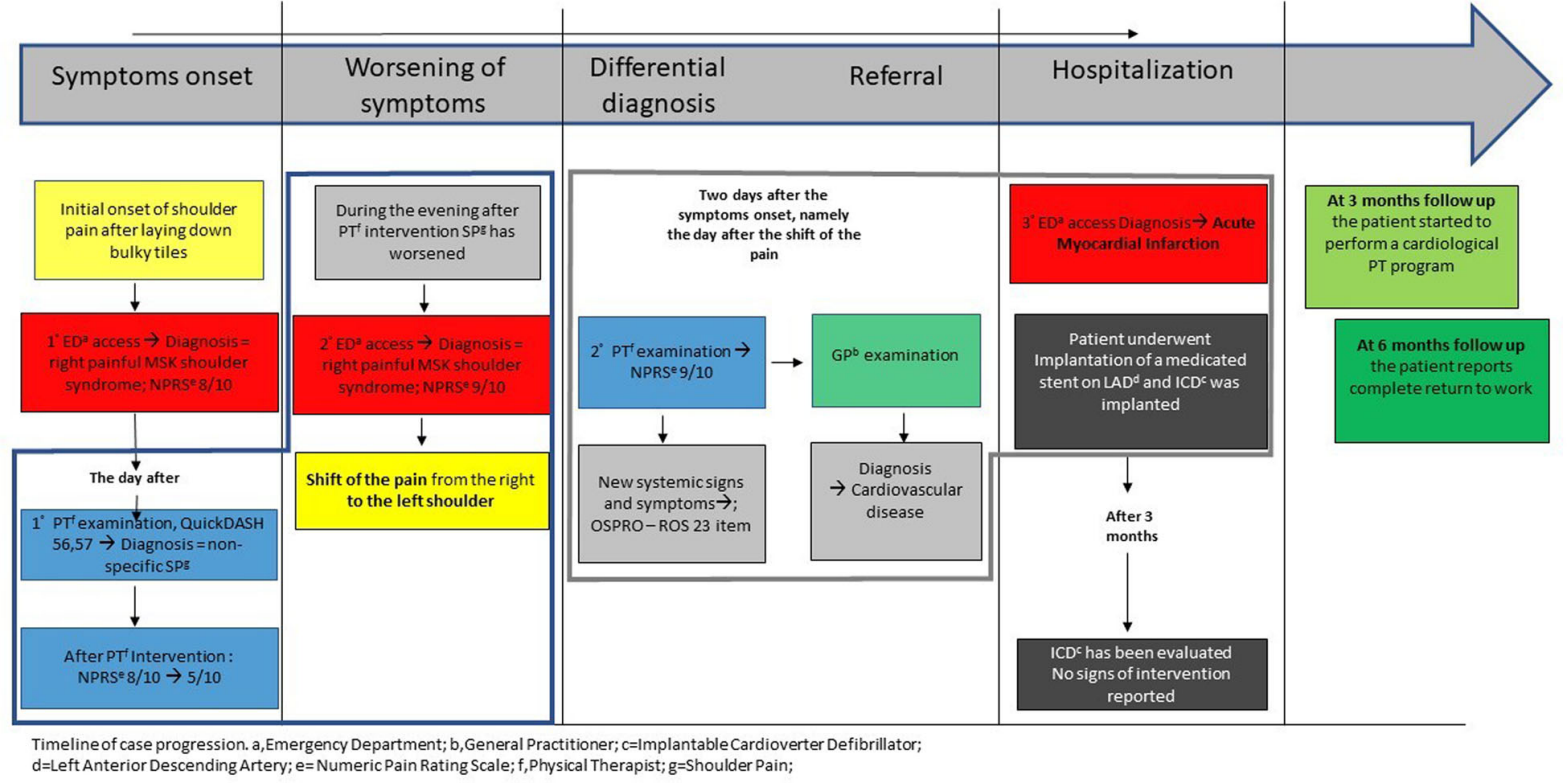
Timeline

After a week of hospitalization in the surgical unit, the heart surgeon decided to subject the patient to the implantation of an implantable cardioverter defibrillator (ICD) due to persistent arrhythmia. At 3 months follow-up, ICD has been evaluated and no signs of intervention were detected. Furthermore, neither signs nor symptoms of SP or viscerogenic disease has been reported. Thereafter, the cardiologist allowed performing a cardiological physiotherapy program, which has been carried out for 4 weeks after the cardiologist prescription in a licensed outpatient clinic for cardiological rehabilitation. The exercise protocol used was similar to the recently published exercise protocol for cardiac rehabilitation of Satyamurthy et al., 202 0[46]. The outcome measures adopted for the evaluation of the patient’s achievements were the 6 min walking test for the endurance performance [47], the Borg scale for self-perceived exertion [48], and the Medical Outcome Study 36-item Short-Form Health Survey [49] to assess the awareness and the quality of life. Finally, at 6 months follow-up, the cardiologist authorized the patient to a complete return to work after reporting a 31/40 value on general perceived self-efficacy scale (GSE )[50]. For a more detailed story management, see the timeline in Fig. 5.

## Discussion and conclusion

Our case report describes the clinical decision making by a PT that led to detect a non-MSK condition in a 46-y-old male with SP symptoms. SP is one of the most frequent musculoskeletal diseases in outpatient clinics and its prevalence in the general population is within 27% in the lifetime [2, 5, 6]. Moreover, as in our case, shoulder pain can be a somatic sign of certain visceral life-threatening pathologies which mimic musculoskeletal disorders of the trunk or the upper limbs [39–41, 51]. Among these, it must be considered AMI [51], which usually refers pain to chest or upper limb [9, 38, 40, 41]. For that reason, physicians and healthcare professionals must be alert in differential diagnosis process during the physical examination of the shoulder [5, 23, 40, 41], especially when palpation of the painful areas does not reproduce the patient symptoms [52], or in presence of autonomic signs as fever, diaphoresis, nausea and respiratory complaints which frequently occurring in presence of heart related visceral pain [7, 53, 54]. Those signs, within musculoskeletal context are defined as Red Flags (RF) (e.g. clinical indicators of possible serious pathologies )[40, 41] and, notwithstanding RFs as self-contained screening tools seem to be weak during the physical examination of the patients with some MSK diseases [52], the combination of multiple RFs for the screening for referral process is promising [55, 56]. Furthermore, clinical decision making in patients with MSK pain conditions can be supported using validated tools [42–45], in order to perform or not a review-of-systems [5, 23] and for medical referral [42–45]. As in this case, direct access to PTs is becoming an increasingly common practice [9], however, not all PT in the world are allowed to prescribe imaging [57], hence, it is critical that PT being able to perform a clinical review-of-systems within differential diagnosis process [40, 41]. For this reason, screening of personal and family medical history, assessment of the risk factors (e.g. smoking, body mass index, congenital or hereditary diseases), screening for RFs [55], watchful waiting [52], and linking RFs symptomology directly with health status and vital parameters, are mandatory professional skills for PT in direct access clinical settings [5, 9, 23], as it has been clearly stated from a widespread clinical practice guideline [58]. From the patient perspective, the physical examination conducted by the PT demonstrated the professional skills needed to decision-making process for differential diagnosis and medical referral. About medication intake in case of suspected cardiovascular diseases, the use of non-steroidal anti-inflammatory drugs (NSAID) as potential aggravating factors in case of AMI are well known [59–63]. Specifically, diclofenac-based medications should be carefully administered under medical prescription and thus avoided as over-the-counter drugs because of high cardiovascular associated risks [60]. Moreover, attention must be paid even to use other NSAID such as ibuprofen or paracetamol, which despite have shown lower risks of cardiovascular events than diclofenac [59–61], pose a substantial aggravating risk in case of AMI. Conversely, acetylsalicylic acid drugs shall be mentioned in this context because their use under suspicion of AMI must be encouraged in preventing major cardiovascular diseases [64, 65]. The shift in symptom from the right to the left shoulder is an unusual presentation of AMI within clinical scenario, however the array of symptoms that could be referred by patients after an AMI is multiple [8]. Among them, pain in one or both shoulders seem to be correlated to the site of AMI [66]. The network underlying this phenomenon is related to viscerosomatic convergence onto upper thoracic spinothalamic tract neurons. In fact, both cardiac sensory information and somatic sensory information from the chest and upper limb merge onto the same bunch of spinothalamic tract neurons in the upper thoracic (T1-T5) spinal dorsal horn segments [67]. Consequently, this convergence does not allow to project the incoming information accurately onto the somatosensory cortex [68]. This mechanism leads to overlapped pathways for somatic sensations, and besides it has been further tested in a recent case report in which a 29 male patient referred intermittent crushing substernal chest pain localized to the left lateral sternal border which radiated to the left arm following vagal nerve stimulation [69]. Moreover, as for what the neurophysiology is concerned, tissue trauma may result in an afferent barrier of nociceptive transmission that could alter the threshold for excitation stimuli in both central and peripheral areas, enhancing the afferent transmissions to the dorsal horn of the spinal cord, thus expanding the receptive field of dorsal horn neurons, which is called central hypersensitization [70, 71]. The patient in the present case report has started to feel shoulder symptoms on the right side, which had been referred to as the site of several dislocations in childhood. Conversely, the pain in the left shoulder and the pectoral region showed up only 9 days after the initial symptom onset. Furthermore, the left shoulder pain has started concurrently with the autonomic symptoms as dyspnea, fatigue, shortness of breath and diaphoresis which has been recently linked to specific sites of myocardial infarction [72]. In this perspective, the progression of the hypoxic injuries induced in the myocardium following an inadequate exposure to oxygen supply could have affected specific sympathetic fibers, that even provoked the shift in somatic pain as well. However, as matters stand at present, there is not sufficient evidence to enable us to state which of the above mechanisms is actually responsible for the development of the shift in symptom from the right to the left shoulder.

To the best of the authors’ knowledge, this is the first documented case of AMI that has been manifested through an upper limb MSK pain pattern recognized by a PT. Such an unusual clinical presentation, with a sudden shift from right to the left side with new signs and symptoms, allowed the detection of cardiovascular disease during physical examination. This case report highlights the importance of the clinical reasoning during differential diagnosis process by healthcare professional within musculoskeletal context.

## Data Availability

All data generated or analyzed during this care report are included in this published study. Other information of this case report are available from the corresponding author on reasonable request.

## Abbreviations

AMI: Acute Myocardial Infarction
BP: Blood Pressure
ECG: Electrocardiography
ED: Emergency Department
GSE: General perceived self-efficacy scale
HR: Heart Rate
ICD: Implantable Cardioverter Defibrillator
LAD: Left anterior Descending Artery
MRC: Medical Research Council scale
MSK: Musculoskeletal
NPRS: Numeric Pain Rating Scale
PT: Physiotherapist
RF: Red Flag
ROM: Range of Motion
RR: Respiratory Rate
SLAP: Superior Labral Tear from Anterior to Posterior
SP: Shoulder Pain
NSAID: Non-Steroidal Anti-Inflammatory Drugs
